# Preparation, pharmacokinetics and anti‐obesity effects on dogs of nuciferine liposomes

**DOI:** 10.1002/vms3.70017

**Published:** 2024-09-06

**Authors:** Jiang Lu, Yi‐Tian Xu, Xiao‐Liang Qian, Dao‐Xian Zhu, Jin‐Ye Lu, Hui Ma, Jing Liu

**Affiliations:** ^1^ Department of Pet Science and Technology Jiangsu Agri‐Animal Husbandry Vocational College Taizhou China; ^2^ Department of Canine Disease Outpatient Wuxi Paideshi Pet Hospital Wuxi China; ^3^ Department of Animal Medicine Jiangsu Agri‐Animal Husbandry Vocational College Taizhou China

**Keywords:** anti‐obesity, dogs, in vitro release, liposome, nuciferine, pharmacokinetics

## Abstract

**Background:**

Nuciferine (NUC), a natural compound extracted from lotus leaves, has been proven to have anti‐obesity effects. However, the development and application of NUC as an anti‐obesity drug in dogs are hindered due to its poor water solubility and low bioavailability.

**Objective:**

To promote the development of NUC‐related products for anti‐obesity in dogs, this study prepared NUC into a liposome formulation and evaluated its characteristics, pharmacokinetics in dogs, and anti‐obesity effects on high‐fat diet dogs.

**Methods:**

NUC liposomes were prepared by the ethanol injection method, using NUC, egg lecithin, and β‐sitosterol as raw materials. The characteristics and release rate in vitro of liposomes were evaluated by particle size analyser and dialysis method, respectively. The pharmacokinetics in dogs after oral administration of NUC‐liposomes was carried out by the high‐performance liquid chromatography (HPLC) method. Moreover, we investigated the anti‐obesity effect of NUC‐liposomes on obese dogs fed with a high‐fat diet.

**Results:**

NUC‐liposome was successfully prepared, with an EE of (79.31 ± 1.06)%, a particle size of (81.25 ± 3.14) nm, a zeta potential of (–18.75 ± 0.23) mV, and a PDI of 0.175 ± 0.031. The cumulative release rate in vitro of NUC from NUC‐liposomes was slower than that of NUC. The *T*_1/2_ and relative bioavailability of NUC‐liposomes in dogs increased, and CL reduced compared with NUC. In addition, the preventive effect of NUC‐liposomes on obesity in high‐fat diet dogs is stronger than that of NUC.

**Conclusions:**

The liposome formulation of NUC was conducive to improve its relative bioavailability and anti‐obesity effect in dogs.

## Graphical abstract

1. Nuciferine (NUC) has an anti‐obesity effects. However, its low bioavailability hinders the application of NUC.

2. NUC‐Liposomes made by encapsulating NUC with egg yolk lecithin and β‐sitosterol extended the half‐life of NUC, reduced clearance rate and improved the relative bioavailability of NUC in dogs.

3. NUC‐liposomes significantly reduced the body weight of high‐fat diet induced obese dogs compared with free NUC, suggesting NUC‐liposomes as potential dosage form for preventing canine obesity.

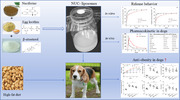

## 1 INTRODUCTION

**FIGURE 1:**
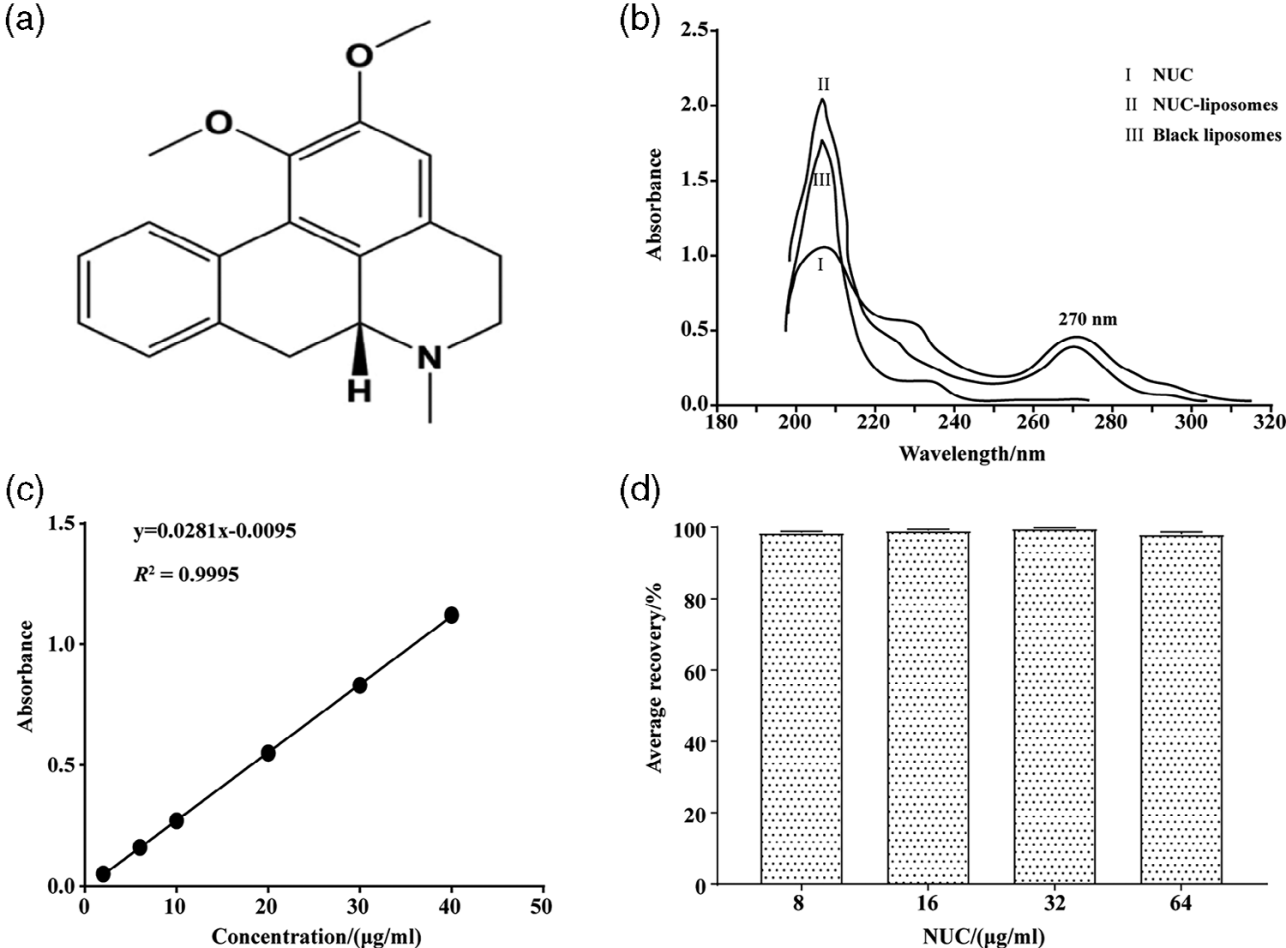
Standard curve of NUC using UV absorption method.

Nuciferine (NUC, Figure 1A) is the main biologically active ingredient obtained from the dried leaves of *Nelumbo nucifera* Gaertn (Huang et al., 2022; Sharma et al., 2017) and has been reported to possess extensive pharmacological activities, including anti‐obesity (Dai et al., 2016), anti‐atherosclerosis (Xiao et al., 2022), anticancer (Kang et al., 2017), anti‐inflammatory (Wu et al., 2017) and antioxidation (Liu et al., 2014). NUC could exert anti‐obesity effects through multiple pathways and targets, such as inhibition of lipogenesis via activating AMPK signalling pathway (Ma et al., 2015) and regulating Akt‐mTORC1 signalling pathway (Yoo et al., 2019) and decrease of intracellular lipid accumulation by modulating the expression of lipogenic genes and adipokines (Xu et al., 2021). Recently, some studies showed that the anti‐obesity effect of NUC was performed by means of modulating the composition and potential function of the gut microbiota (Shi et al., 2021; Wang et al., 2020; Xiong et al., 2021; Yu et al., 2021). Moreover, there are few reports regarding the toxicity of NUC. However, a study has shown that NUC affected the liver concentration and hypoglycaemic effect of metformin for a while (30 min and 60 min) when NUC was used in combination with metformin (Li et al., 2018), suggesting the potential risks associated with NUC combination therapy. Although NUC has tremendous potential in the development of anti‐obesity drugs, the literature on the evaluation of the anti‐obesity effect of NUC in dogs is few. In our previous research, we added NUC to the feed and evaluated its weight loss effect on obese dogs. However, the experimental results showed that significant weight loss was not observed. To our knowledge, the low bioavailability of NUC may limit its weight loss effect. The results of a pharmacokinetic experiment on oral administration of different doses of NUC in beagles showed that the times of *t*_1/2ka_ (rang: 0.38–0.44 h), *t*_1/2α_ (rang: 0.22–0.26 h), and *t*_1/2β_ (rang: 0.56–0.85 h) were very short (Wang et al., 2008), suggesting that the rapid absorption, metabolism, and elimination of NUC are the main factors for its low bioavailability. In addition, the low water solubility of NUC can reduce its bioavailability as well. It is necessary to improve the bioavailability of NUC through some methods. Previous study showed that co‐loaded with NUC and epigallocatechin‐3‐gallate of microgel improved the stability and oral bioavailability of NUC and reduced the body weight of high‐fat diet rats (Zhu et al., 2022).

The development and application of many natural compounds, including NUC, are impeded because of their low bioavailability. Fortunately, numerous new dosage forms for improving bioavailability of these natural compounds have been very successfully developed, such as microcapsules (Zhang et al., 2021), nanoparticles (Kim et al., 2018), solid dispersions (Tran & Tran, 2020) and liposomes (Wang et al., 2022a). Among these dosage forms, the unique advantages of liposomes mainly include biocompatibility and biodegradability, increased stability and water solubility, and increased cell membrane permeability (Barenholz, 2003; Bozzuto & Molinari, 2015). Hence, we hypothesised that NUC‐liposomes might improve the bioavailability of NUC and enhance its anti‐obesity effect on obese dogs.

In the present study, NUC‐liposomes were prepared using ethanol injection method and the preparation process was optimised using response surface methodology. Then, the relevant characteristics of the obtained liposomes were evaluated. Furthermore, the pharmacokinetics of NUC‐liposomes after single oral administration in beagles was estimated by determination of the concentration of NUC in serum using the HLPC method. Moreover, we estimated the anti‐obesity effects of NUC‐liposomes supplementation in high‐fat diet‐induced obese beagles. This research may provide support for the application of NUC in the prevention and treatment of canine obesity.

## 2 MATERIALS AND METHODS

### 2.1 Materials and reagents

The standard NUC (purity > 99%) was obtained from the China Institute for the Control of Food and Drug Products (Beijing, China). Egg lecithin (purity > 99%) was purchased from Shanghai Zhanyun Chemical Co., Ltd (Shanghai, China). β‐sitosterol (purity>98%) was supplied from Shanghai Yeyuan Biotechnology Co., Ltd (Shanghai, China). All other chemicals were of analytical grade.

### 2.2 Animals and diet

Healthy adult male beagles (average age: 3.5 ± 0.2 years, and average body weight: 10.25 ± 0.87) were provided by Jiangsu Taist Biotechnology Co., Ltd (Taizhou, China; Production License for Experimental Animals: SCXK (Su) 2021‐0010). All experiments involving dogs were performed using protocols approved by Institutional Animal Care and Use Committee of Jiangsu Agri‐animal Husbandry Vocational College (NSF202108), China and the procedures were carried out following Regulations on the Management of Experimental Animals, China (Directive 1988/2/China) for the care and use of experimental animals.

Before the beginning of the experiment, the experimental dogs were subjected to physical examinations, complete blood count using the haematology analyser (ProCyte Dx, Idexx Laboratories, Maine, USA), blood biochemical analysis using the blood biochemistry analyser (BS‐240Vet, Shenzhen Mindray Biomedical Electronics Co., Ltd, Shenzhen, China) and urinalysis using urine analyser (BT200, Shengshi Dongtang Jiangsu Biotechnology Co., Ltd, Taizhou, China). The results showed that all detection indicators were within the reference range, and the body weight was in an ideal state (BCS using a 9‐point BCS system was 5), indicating that these dogs were healthy.

Normal diet (ND, 10% calories from fat) and high‐fat diet (HFD, 55% calories from fat) were purchased from Nantong Trophy Animal Feed Technology Co., Ltd (Nantong, China).

### 2.3 Preparation of NUC‐liposomes

The ethanol injection method was used to prepare NUC liposomes. NUC (5.0 mg), egg lecithin (50 mg) and β‐sitosterol (10 mg) were completely dissolved in anhydrous ethanol (10 mL). The ethanol mixture obtained was injected into normal saline (30 mL) at 45°C and used a magnetic stirrer to stir at 600 r/min for 30 min. Then, the ethanol and a part of water are removed by rotating evaporation at 45°C. Finally, NUC‐liposomes were obtained after dialysis with PBS solution (pH 5.5) and filtered through membrane filtration (0.22 μm). In addition, blank liposomes were prepared as well.

### 2.4 Encapsulation efficiency of NUC‐liposomes

The standard NUC was dissolved in methanol solution to prepare standard NUC solution (100 μg/mL). The standard NUC solution (0.5 mL), blank liposomes (0.5 mL) and NUC‐liposomes were diluted to 5 mL using methanol solution, respectively. Diluted standard NUC solution, blank liposome supernatant, and NUC‐liposomes, with blank solvent as control, were scanned using a UV visible spectrophotometer in the range of 180–400 nm to determine the maximum absorption wavelength of lotus leaf alkaloids and whether NUC‐liposomes have been successfully prepared.

Then, we accurately extracted 0.1, 0 3, 0.5, 1.0, 1.5 and 2.0 mL of standard NUC solution (100 μg/mL), diluted them to 5 mL with methanol solution, shook well, and measured the absorbance at 270 nm using UV visible spectrophotometer. Each sample was repeated 3 times to take the average value. The standard curve of NUC was obtained through linear regression with the sample concentration as the *x*‐axis and the absorbance value as the *y*‐axis. The accuracy of the standard curve was evaluated by measuring the recovery rate of standard NUC solution (8, 16, 32 and 64 μg/mL).

Encapsulation efficiency (EE) was calculated using the equation: EE (%) = (quality of NUC in liposomes/quality of total NUC) × 100%. The measurement process of the quality of the drug in liposomes was as follows: NUC‐liposomes suspension (0.5 mL) diluted to 5 mL with methanol solution. After 200 W ultrasonic demulsification, the absorbance at 270 nm was measured using a UV visible spectrophotometer. The NUC content was calculated based on the standard curve and then converted to mass. The quality of total NUC was the input mass during liposome preparation.

### 2.5 Optimisation of the preparation process

Based on the single‐factor experiments, a three‐factor Box–Behnken design at three levels was carried out to screen the main effects of the three factors on response to EE. Design Expert software (version V13.0) was used for experimental design and data analysis. The range and the levels of experimental variables in this study are presented in Table 1.

**TABLE 1:** Codes and levels for NUC‐ liposome preparation.

Variables	Code	−1	0	1
Egg lecithin/ β‐sitosterol ratio (g/g)	*X*_1_	4∶1	6∶1	8∶1
Drug/lipid ratio (g/g)	*X*_2_	1∶8	1∶12	1∶16
Ethanol/normal saline ratio (v/v)	*X*_3_	1∶1	1∶2	1∶3

### 2.6 Characterisation of NUC‐liposomes

The particle size, zeta potential and polydispersity index (PDI) of NUC‐liposomes were measured using Particle Size Analyzer (Nano‐ZS, Malvern Instruments, Malvern, Britain). The samples were diluted 10‐fold with distilled water, and three batches of samples were measured.

### 2.7 Release behaviour of NUC‐liposomes in vitro

NUC and NUC‐liposomes were put into dialysis bags (with a molecular weight of 6–8 KD) separately. The dialysis bags were then immersed in dissolution flasks containing 100 mL of PBS (pH 5.5 and pH 7.4) solution, respectively. The entire flask was immersed in a constant temperature bath set at 37°C with a paddle speed of 50 rpm. Finally, 2 mL of release medium was removed at a specified time (0.5, 1, 1.5, 2, 4, 6, 8, 12, 18, 24, 36 and 48 h) and immediately replaced with an equal volume of fresh release medium. The quantity of oridonin was determined using a UV visible spectrophotometer at 270 nm, and the cumulative release percentage (CR) of NUC was calculated using the equation: CR(%) = (C*_n_* × *V* + Σ*C_i_* × *V_s_*) /*Q*_0_ × 100. In the equation, *C_n_* is the concentration of NUC at sampling point *n*, *C_i_* is the concentration of NUC at the *i* sampling point, *V* and *V_s_* are 100 mL and 1 mL respectively, and *Q*_0_ is the initial quality of NUC. The experiment was repeated 3 times.

### 2.8 Pharmacokinetic study of NUC‐liposomes in vivo

#### 2.8.1 Establishment of NUC standard curve

The concentration of NUC was determined by HPLC method. Briefly, the standard sample of NUC was accurately weighed at 2.00 mg and dissolved in methanol to a volume of 2 mL in a volumetric flask. Then, the NUC working solutions with concentrations of 0.5, 1, 5, 20, 100 and 200 ng/mL were diluted with methanol for testing. Chromatography conditions of HPLC were as follows: Sinochrom ODS‐BP column (250 mm × 4.6 mm, 5 μm). The mobile phase consisted of acetonitrile, water, glacial acetic acid and triethylamine (40:60:0.5:0.43) with a flow rate of 1.2 mL/min and column temperature of 30°C. A standard curve (0.5–200 ng/mL) with standard concentration was drew as the horizontal axis and peak area as the vertical axis.

#### 2.8.2 pharmacokinetic evaluation

Male beagles were randomly divided into two groups, six in each group, and fasted for 12 h before the experiment, with free drinking water. NUC (a single dose of 10 mg per kilogram of body weight) and NUC‐liposomes (a single dose of 125 mg per kilogram of body weight, equivalent to 10 mg/kg NUC) were orally administrated to the beagles. 2.0 mL blood sample was drawn through the lateral radial vein at time intervals of 0.5, 1, 1.5, 2, 4, 6, 8, 12 and 24 h after administration, centrifuged (4000 r/min, 10 min), collected the plasma and stored at −20°C. Methanol (1.8 mL) was added to 200 μL plasma, vortexed and mixed for 3 min, and centrifuged for 10 min (10,000 r/min). The supernatant was blow‐dried with nitrogen at room temperature. The residue was redissolved in 200 μL methanol, vortexed for 2 min, and centrifuged for 10 min (10,000 r/min). The supernatant was detected by HPLC.

The analysis of pharmacokinetic parameters was conducted using a noncompartmental model, and the peak concentration (*C*_max_), peak time (*T*_max_), half‐life (*T*_1/2_), mean residence time (MRT), total clearance (CL), apparent volume of distribution (Vd), area under the concentration time curve (AUC) and relative bioavailability (*F*) were calculated using Phoenix WinNonlin software (version 6.3, Pharsight, California, USA).

### 2.9 Preventive effects of NUC‐liposomes on obesity in dogs

#### 2.9.1 Experimental design

After 2 weeks of adaptive feeding with a normal diet, a total of 24 beagles were randomly divided into 4 groups: normal diet group (ND), high‐fat diet group (HFD), HFD + NUC group (HFD+NUC), and HFD+ NUC‐liposomes group (HFD+NUC‐L). The ND group was fed a normal diet, while the other three groups were fed with HFD. The animals were kept in specialised animal rooms with an ambient temperature of (25 ± 2)°C and an indoor humidity of around 60%. Animals are fed twice per day (at 8:30 and 16:30, each time for 10 min, with ad libitum water drinking). In addition, the HFD+NUC group were orally administered with NUC at a dose of 10 mg/kg body weight, while the HFD+NUC‐L group were orally administered with NUC‐liposomes at a dose of 125 mg/kg body weight (equivalent to 10 mg/kg NUC), twice per day (at 8:00 and 16:00), respectively. The ND and HFD groups were orally administered with blank‐liposomes at a dose of 125 mg/kg body weight, respectively. The experimental period was 8 weeks. The feeding weight and remaining weight of the diet were observed every day, and the daily feed intake (DFI) was calculated. The average daily feed intake (ADFI) was calculated using the equation: ADFI (g/d) = DFI/days. The body weight of dogs was measured at 0, 2, 4, 6 and 8 weeks. At the end of the experiment, blood samples were collected from the lateral radial vein. The serum was extracted by centrifugation of the blood samples at 3000 rpm for 15 min at 4°C and stored at −80°C for subsequent analysis. After the experiment, the body weight of all obese dogs returned to normal through diet and exercise.

#### 2.9.2 Subcutaneous fat thickness measurement

An ultrasound diagnostic instrument (model: MyLab 9, Esaote Company, Italy) was used to scan the posterior part of the umbilical foramen in the dog's abdomen using a 7.5 MHZ linear array probe before the beginning of the experiment (0 day) and at the end of the experiment (56 days), respectively. The subcutaneous fat thickness was measured based on B‐ultrasound images. The subcutaneous fat thickness was the average of the three measurements.

#### 2.9.3 Biochemical analysis

The serum levels of triglyceride (TG) and total cholesterol (TC) were measured by using the Catalyst Dx automatic biochemical analyser with the corresponding commercial kits (IDEXX Laboratories, Inc., Maine, USA).

### 2.10 Statistical analysis

Data are presented as mean ± SD. Statistical analyses were performed using SPSS 23.0 software. Statistical differences were analysed by one‐way ANOVA followed by an LSD test. Paired sample *t*‐test was used for haematological and serum biochemical parameters between before and after treatment with NUC‐liposomes. The statistically significant difference was set to *p* < 0.05.

## 3 RESULTS

### 3.1 Preparation of NUC‐liposomes

#### 3.1.1 Standard curve of NUC

Figure 1 shows the UV absorption characteristics and standard curve of NUC. The absorption peak was observed at 270 nm for NUC and NUC‐liposomes, while the blank liposomes showed no absorption peak at 270 nm, indicating that the absorption peak at 270 nm is unique to NUC and preparation of NUC‐liposomes is successful as well (Figure 1b). The standard curve of NUC was *y* = 0.0281*x* – 0.0095 (*R* ^2^ = 0.9995, Figure 1c). Based on this standard curve, the average recovery rates of NUC (*n* = 3) were 98.53%, 99.12%, 99.67% and 98.07% at concentrations of 8, 16, 32, and 64 μg/mL, respectively (Figure 1d). These results indicated that UV absorption method to calculate EE of NUC liposomes was feasible.

#### 3.1.2 Optimisation design of preparation

The results of three factors and three levels of Box–Behnken design and response variables (EE) are presented in Table 2. By applying multiple regression analysis on the experimental data, the regression equation between the independent variable and the response value was as follows: *Y* = 78.93 – 1.54*X*_1_ – 1.27*X*_2_ + 0.59*X*_3_ + 0.67*X*_1_ *X*_2_ – 0.15*X*_1_ *X*_3_ + 0.57*X*_2_ *X*_3_ – 5.22*X*_1_^2^ – 7.26*X*_2_^2^ – 0.58*X*_3_^2^. Analysis of ANOVA results for the response surface quadratic model is presented in Table 3. The *p*‐value of lack of fit (*p* = 0.5349) was insignificant, suggesting pure error was less. The low *p*‐value of the model (*p* < 0.0001) indicated that the models were significant and could be used to predict the actual experimental data.

**TABLE 2:** Box–Behnken design and experimental encapsulation efficiency (EE).

	*X*_1_	*X*_2_	*X*_3_	EE/%
1	−1	0	−1	73.48
2	0	0	0	77.00
3	0	1	1	70.47
4	0	0	1	80.22
5	−1	−1	0	70.33
6	1	−1	0	65.47
7	0	0	0	79.93
8	0	−1	−1	72.54
9	0	0	0	79.43
10	0	0	0	78.74
11	0	1	−1	68.89
12	−1	1	0	66.41
13	1	1	0	64.24
14	0	−1	0	71.84
15	−1	0	1	75.08
16	1	0	1	72.15
17	1	0	−1	71.16

**TABLE 3:** Results of ANOVA analysis.

		EE	
Effects	Source model	*F*‐value	*p* value
Model		39.23	<0.0001
Linear	*X*_1_	39.23	<0.0001
	*X*_2_	16.67	0.0047
	*X*_3_	11.43	0.0117
Interaction	*X*_1_ *X*_2_	2.74	0.1419
	*X*_1_ *X*_3_	1.6	0.2464
	*X*_2_ *X*_3_	0.082	0.7826
Quadratic	*X*_1_^2^	1.15	0.3193
	*X*_2_^2^	101.15	< 0.0001
	*X*_3_^2^	195.16	< 0.0001
	Lack of fit	0.85	0.5349

**FIGURE 2:**
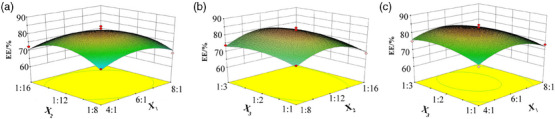
Response surface plots representing the effect of process conditions on EE. (a) The effect of *X*_1_ and *X*_2_ on EE; (b) the effect of *X*_2_ and *X*_3_ on EE; and (C) the effect of *X*_1_ and *X*_3_ on EE.

Figure 2 shows the 3D response surface plot of factors and EE. The EE showed a trend of first increasing and then decreasing when all three factors(*X*_1_, *X*_2_ and *X*_3_) increased. Optimisation of liposome preparation was based on higher EE. Using regression models for prediction analysis, the EE of NUC‐ liposomes was 79.25% when the optimal preparation conditions of *X*_1_, *X*_2_ and *X*_3_ were 5.7:1, 1:11.5 and 1:2.5, respectively.

#### 3.1.3 Characteristics of NUC‐liposomes

The confirmatory experiment (*n* = 3) was performed under optimised conditions: egg lecithin/ β‐sitosterol ratio of 5.7:1, drug/lipid ratio of 1:11.5, and ethanol/normal saline ratio of 1:2.5. The experimental EE was (79.31 ± 1.06)%, which is consistent with the predicted EE of 79.25%. The particle size of NUC‐liposomes was (81.25 ± 3.14) nm, the electric potential was (−18.75 ± 0.23) mV, and the PDI was 0.175 ± 0.031, manifesting that the NUC‐liposomes were evenly distributed and had strong stability.

### 3.2 In vitro release study

**FIGURE 3:**
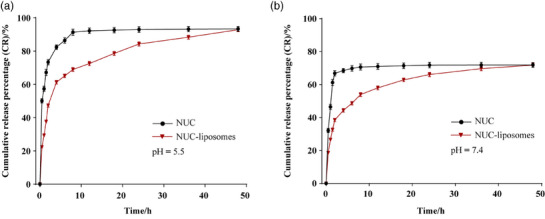
In vitro cumulative release of NUC.

The cumulative release percentage (CR) of NUC measured by dialysis membrane diffusion method is shown in Figure 3. At weak acid condition (pH 5.5), the CR of free NUC after 8 h was 92.36%, while the CR of NUC from liposomes was 68.96% (Figure 3a). However, the CR of free NUC and NUC from liposomes after 8 h was comparatively low (70.64% and 59.93%, respectively) at pH value of 7.4 (Figure 3b). The in vitro release of Nuc liposomes fitted by the in vitro release model conformed to the first‐order release model and has a correlation (*R* ^2^ = 0.9914).

### 3.3 Pharmacokinetic study

**FIGURE 4:**
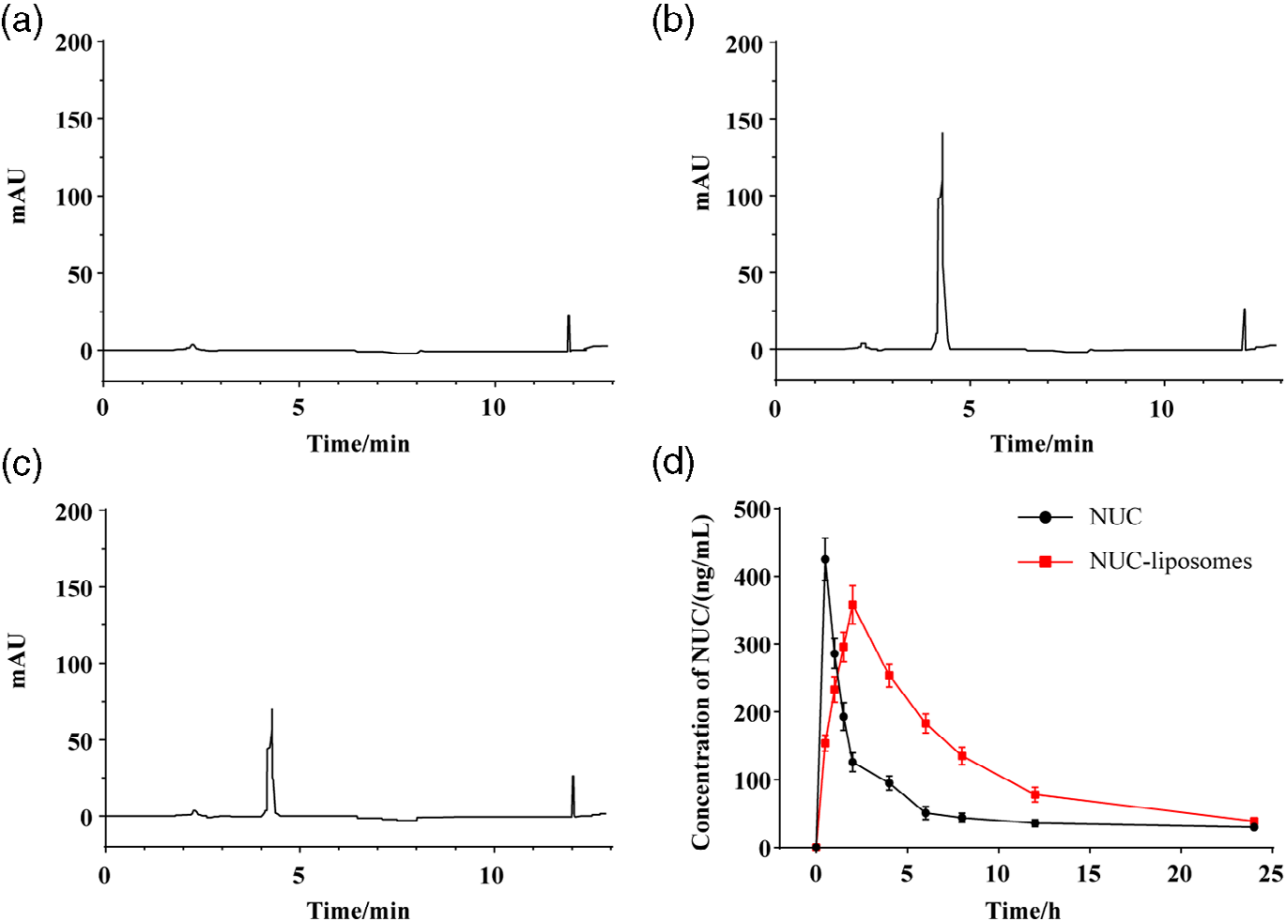
Chromatogram and plasma drug concentration of NUC.

Figure 4 shows the chromatogram of the plasma NUC concentration after oral administration in dogs. The pharmacokinetic parameters are shown in Table 4. The results showed that the relative bioavailability of NUC‐liposomes was 1.96 times than that of NUC. Compared with NUC, the *T*_max_ (2.02 ± 0.13 h vs. 0.51 ± 0.03 h), *T*_1/2_ (6.46 ± 0.56 h vs. 3.15 ± 0.49 h), MRT(0–24) (9.53 ± 0.79 h vs. 4.38 ± 0.53 h), Vd (31.37 ± 4.04 L/kg vs. 13..81 ± 1.48 L/kg), and AUC(0–24) (2926.13 ± 205.23 μg·h/L vs. 1501.50 ± 163.27 μg·h/L) of NUC‐liposomes were significantly increased, while the *C*_max_ (356.66 ± 27.55 μg/L vs. 425.42 ± 44.15 μg/L) and CL (3.42 ± 0.84 L/h/kg vs. 6.58 ± 0.71 L/h/kg) were reduced.

**TABLE 4:** Pharmacokinetic parameters of UNC and NUC‐liposomes.

Parameters	NUC	NUC‐liposomes
*C*_max_/(μg/L)	425.42 ± 44.15	356.66 ± 27.55^*^
*T*_max_/h	0.51 ± 0.03	2.02 ± 0.13^*^
*T*_1/2_/h	3.15 ± 0.49	6.46 ± 0.56^*^
MRT(0–24)/h	4.38 ± 0.53	9.53 ± 0.79^*^
CL/(L/h/kg)	6.58 ± 0.71	3.42 ± 0.84^*^
Vd/(L/kg)	13..81 ± 1.48	31.37 ± 4.04^*^
AUC(0–24)/(μg·h/L)	1501.50 ± 163.27	2926.13 ± 205.23^*^
*F*/%		196.64

* *p* < 0.05 versus NUC.

### 3.4 Preventive effects of NUC‐liposomes on obese dogs

**FIGURE 5:**
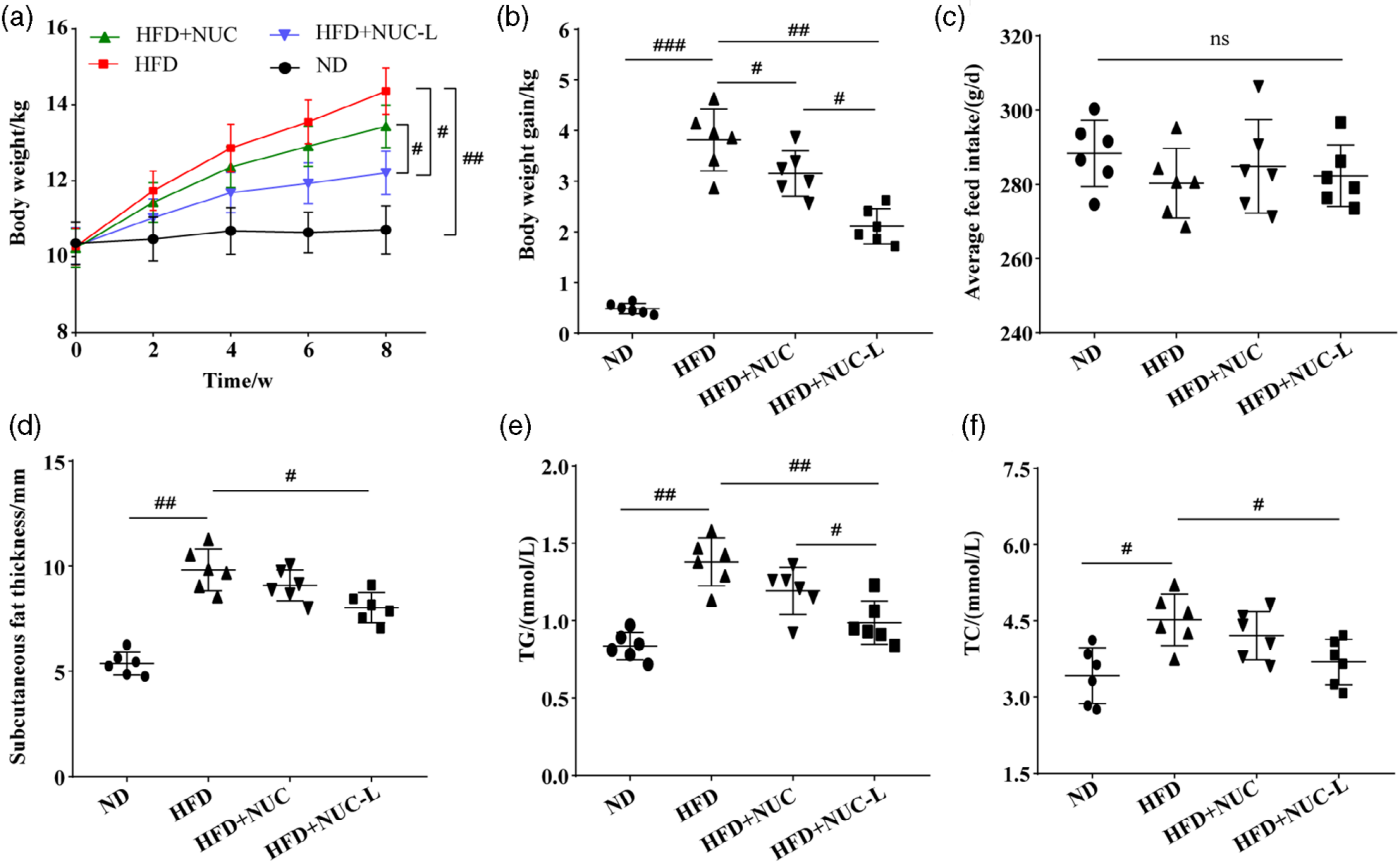
NUC‐liposomes prevents HFD‐induced obesity in dogs.

An obese dog model was established by feeding dogs an HFD for 8 weeks. HFD feeding for this period led to significant increases in body weight and subcutaneous fat thickness compared with the ND group (Figure 5a, b and d). NUC or NUC‐ liposomes supplementation significantly decreased body weight in HFD‐fed dogs (Figure 5a and b). Moreover, supplementing NUC‐liposomes significantly decreased subcutaneous fat thickness in HFD‐fed dogs (Figure 5d). Significantly, the body weight in the HFD+NUC‐L group was lower than those in the HFD+NUC group (Figure 5a and b). Average feed intake had no statistically significant differences between groups (Figure 5c). Compared with the HFD group, the serum TG in the HFD+NUC group and the serum TG and TC in the HFD+NUC‐L group were significantly decreased, and the serum TG in the HFD+NUC‐L group was lower than that in the HFD+NUC group (Figure 5e and f). These results indicate that NUC‐liposomes have a more outstanding anti‐obesity effect on high‐fat diet dogs than NUC. Haematological parameters and serum biochemical parameters of high‐fat diet dogs treated with NUC‐liposomes are presented in Tables 5 and 6, respectively. There were no significant differences in haematological and serum biochemical parameters between before and after treatment with NUC‐liposomes. Furthermore, no adverse reactions were observed during the experiment period.

**TABLE 5:** Haematological parameters of high‐fat diet dogs treated with NUC‐liposomes.

	ND group		HFD group		HFD+NUC group		HFD+NUC‐L group	
Parameters	0 day	56 days	0 day	56 days	0 day	56 days	0 day	56 days
Red blood cells (×10^12^/L)	6.8 ± 1.2	6.6 ± 1.4	6.6 ± 1.6	6.4 ± 1.4	6.7 ± 1.8	6.9 ± 1.4	6.2 ± 1.1	6.4 ± 1.6
Haemoglobin (g/L)	15.2 ± 2.5	15.4 ± 2.5	14.8 ± 2.4	14.5 ± 2.7	14.4 ± 1.9	14.9 ± 2.5	14.2 ± 1.9	14.5 ± 2.1
HCT (%)	39.7 ± 4.1	39.5 ± 5.2	39.8 ± 4.5	39.4 ± 4.5	38.8 ± 3.5	38.6 ± 3.7	38.3 ± 3.5	39.1 ± 4.3
Total WBC (×10^9^/L)	8.4 ± 2.3	8.7 ± 2.6	8.5 ± 2.6	8.9 ± 2.3	8.8 ± 3.3	8.6 ± 3.6	9.2 ± 2.3	8.9 ± 2.7
Platelet (×10^9^/L)	255 ± 57	256 ± 59	250 ± 52	252 ± 55	258 ± 41	254 ± 48	261 ± 67	259 ± 56

**TABLE 6:** Serum biochemical parameters of high‐fat diet dogs treated with NUC‐liposomes.

	ND group		HFD group		HFD+NUC group		HFD+NUC‐L group	
Parameters	0 day	56 days	0 day	56 days	0 day	56 days	0 day	56 days
TBIL (μmol/L)	8.4 ± 1.1	8.6 ± 1.4	7.8 ± 1.4	8.2 ± 1.8	8.1 ± 1.5	8.2 ± 1.3	8.4 ± 1.3	8.4 ± 1.6
ALP (U/L)	72.8 ± 10.1	73.5 ± 11.2	71.9 ± 9.7	74.5 ± 10.2	72.3 ± 10.6	73.3 ± 10.1	70.9 ± 9.1	71.5 ± 8.2
ALT (U/L)	51.6 ± 5.3	50.1 ± 5.5	50.4 ± 4.8	53.7 ± 5.3	52.2 ± 4.7	50.1 ± 5.2	48.4 ± 4.5	50.3 ± 4.8
AST (U/L)	37.2 ± 4.9	38.6 ± 4.3	37.3 ± 4.4	39.0 ± 4.2	38.2 ± 4.3	38.4 ± 4.5	37.3 ± 3.9	38.1 ± 3.5
BUN (mmol/L)	6.6 ± 2.1	6.4 ± 1.9	6.2 ± 1.7	6.5 ± 1.9	6.3 ± 1.3	6.6 ± 1.7	5.9 ± 1.7	5.7 ± 1.9
Creatinine (μmol/L)	71.3 ± 10.7	70.6 ± 10.4	71.8 ± 11.2	73.3 ± 10.6	70.5 ± 9.2	72.4 ± 10.3	70.8 ± 9.2	70.3 ± 9.5

## 4 DISCUSSION

To solve the problem of short half‐life and low bioavailability of NUC in vivo, we prepared NUC‐liposomes by using the ethanol injection method and evaluated the pharmacokinetics and anti‐obesity effects of NUC‐liposomes in dogs. In the present study, NUC‐liposomes with good characteristics were obtained under the conditions of the prescription process of NUC‐liposomes optimised by using the Box–Behnken response surface method. Furthermore, we observed that the in vitro release rate of NUC in NUC‐liposomes slowed down significantly, and the relative bioavailability of NUC in NUC‐liposomes significantly increased in dogs, when compared with those in NUC. Moreover, the HFD+NUC‐L group had significantly lower body weight and serum TG compared with the HFD and HFD+NUC groups, proving that the anti‐obesity effect of NUC liposomes is superior to that of NUC liposomes.

The ethanol injection method is a preferred method for forming liposomes. Compared with other methods, this method not only avoids the use of toxic solvents (e.g. chloroform and methanol) but also produces liposomes with small particle sizes and uniform dispersion (Bai et al., 2014). In addition, this method is easy to operate and has good reproducibility (Justo & Moraes, 2010). Sebaaly et al. (2015) prepared clove essential oil liposomes (EE: 86.6%; particle size: 260 ± 4.8 nm) by ethanol injection method. In this study, the characteristics of NUC‐liposomes were evaluated as well. The results showed that NUC was encapsulated in liposomes with a particle size of 81.25 ± 3.14 nm, an electric potential of −18.75 ± 0.237 mV, and a PDI of 0.175 ± 0.031, indicating that the ethanol injection method is feasible for the production of NUC‐liposomes.

The stability of liposomes can be improved by a certain proportion of cholesterol during the preparation process of liposomes (Bui et al., 2016). Some research has shown that cholesterol can form liquid‐ordered regions on the bilayer after being added to liposomes, while the arrangement of phospholipid molecular layers is mainly disordered when cholesterol is not added (Rappolt et al., 2003). The improvement of the rigidity of liposome membranes, the decrease of membrane permeability, and the regulation of membrane fluidity are related to cholesterol (Nathalie et al., 2018). However, excessive cholesterol intake may lead to hypercholesterolemia, which is a major factor in cardiovascular disease (Takruri & Alkurd, 2014). The β‐sitosterol has a favourable biological activity, which can reduce the absorption of cholesterol and the incidence of cardiovascular diseases (Kobayashi et al., 2008). Moreover, the molecular structure of β‐sitosterol is similar to that of cholesterol. Therefore, we used β‐sitosterol instead of cholesterol to prepare NUC‐liposomes. Our results indicated that this NUC liposome had the characteristics of high encapsulation efficiency and uniform particle size.

Drug solubility and permeability play a key role in the bioavailability of pharmaceutical preparations. The release behaviour in vitro is an important quality control indicator for liposomes (Wang et al., 2022b). Hence, the in vitro drug dissolution was introduced to simultaneously predict the dissolution properties of NUC taking into account pH changes in this study. We found that NUC‐liposomes significantly slowed down NUC release and maintained a longer drug release cycle, and the in vitro release of the NUC‐liposomes conformed to the first‐order release model, suggesting it may predict the release of NUC‐liposomes in animals. Moreover, we observed that the cumulative release rate of NUC in PBS solution (pH 5.5) was higher than that in PBS solution (pH 7.4), which is consistent with the report of Zhao et al (2018). That report showed that the solubility of UNC increased with the decrease pH, suggesting the lower solubility of NUC in the intestine because intestinal juice has a high pH.

Oral drug delivery is the most convenient way of drug administration. However, oral drug delivery is not the optimal way from the perspective of bioavailability. For example, the absolute bioavailability of NUC in rats administered orally was 3.8%−4.2% (Gu et al., 2014). Encapsulation or entrapment of drugs in liposomes results in distinct changes in the pharmacokinetic and pharmacodynamic properties of free drugs (Shu & Wang, 2017). In this study, we investigated the pharmacokinetics of orally administered NUC‐liposomes in dogs. Our results showed that the *T*_max_, *T*_1/2_ and MRT of NUC‐liposomes significantly increased, while CL significantly reduced compared with NUC. In addition, the relative bioavailability of NUC‐liposomes increased by 1.96 times compared to the raw material drug. These results illustrate that NUC‐liposomes can alter the pharmacokinetics of NUC, and thus may improve associated therapeutic effects. Research has shown that liposomes can improve oral bioavailability of drugs through mechanisms such as protection of the drug in the gastrointestinal tract, increasing drug solubility, increasing cellular contact and residence time of the drug, protection of the drug from presystemic metabolism and efflux, diffusion across the mucus layer, and increasing drug transport across the epithelial membrane (Roger et al., 2010; Saremi et al., 2011). However, our main limitation was that the mechanism by which NUC liposomes enhance the relative bioavailability of NUC was not explored; therefore, further study is needed.

Numerous studies have shown that NUC has an anti‐obesity effect. In high‐fat diet‐fed hamsters, NUC could reduce body weight, percentage of inguinal subcutaneous fat, and levels of serum TC, TG and LDL‐C (Guo et al., 2013). However, supplementation of NUC did not significantly reduce body weight and subcutaneous fat thickness of HFD‐fed obese dogs compared with HFD group in this study. The possible explanation is that NUC was not dissolved in organic solvents before oral administration, resulting in a decrease absorption of NUC and thus weakening its biological activity. Interestingly, oral supplementation of NUC‐liposomes significantly reduced body weight, subcutaneous fat thickness, and the level of serum TG and TC with HFD group in this study, which might be related to the pharmacokinetic characteristics of NUC liposomes. In addition, there were no adverse reactions observed with NUC liposomes. Thus, it is hopeful that NUC‐liposomes will develop into a new type of anti‐obesity product for dogs.

## 5 CONCLUSION

In the present study, we successfully developed NUC‐liposomes with uniform particle size using NUC, egg lecithin and β‐ sitosterol by the ethanol injection method. The results of the in vitro release experiment demonstrated that the slow‐release effect of NUC‐liposomes was significantly improved, while the pharmacokinetic experiment indicated that NUC‐liposomes displayed much higher bioavailability than crude NUC. Furthermore, the anti‐obesity effect of NUC‐liposomes was more outstanding than that of crude NUC in HFD‐fed obese dogs. In summary, this study provides important experimental evidence for the development of NUC‐liposomes as potential drugs for preventing canine obesity.

## AUTHOR CONTRIBUTIONS

**Jiang Lu**: Conceptualisation; project administration; software; writing—original draft. **Yi Xu**: Data curation, resources. **Xiao Qian**: Investigation, resources. **Dao Zhu**: Investigation, methodology. **Jinye Lu**: Writing—review & editing. **Hui Ma**: Supervision. **Jing Liu**: Resources.

## CONFLICT OF INTEREST STATEMENT

The authors declare no conflict of interest.

## INSTITUTIONAL REVIEW BOARD STATEMENT

The study was approved by the Institutional Animal Care and Use Committee of Jiangsu Agri‐animal Husbandry Vocational College (NSF202108).

### ETHICS STATEMENT

All animal experiments were conducted in accordance with guidelines of Institutional Animal Care and Use Committee of Jiangsu Agri‐animal Husbandry Vocational College.

### PEER REVIEW

The peer review history for this article is available at https://www.webofscience.com/api/gateway/wos/peer‐review/10.1002/vms3.70017.

## Data Availability

The data that support the findings of this study are available from the corresponding author upon reasonable request.
